# Low-Cost Light-Based GelMA 3D Bioprinting via Retrofitting: Manufacturability Test and Cell Culture Assessment

**DOI:** 10.3390/mi14010055

**Published:** 2022-12-25

**Authors:** Juan Enrique Pérez-Cortez, Víctor Hugo Sánchez-Rodríguez, Salvador Gallegos-Martínez, Cristina Chuck-Hernández, Ciro A. Rodriguez, Mario Moises Álvarez, Grissel Trujillo-de Santiago, Elisa Vázquez-Lepe, J. Israel Martínez-López

**Affiliations:** 1 Tecnologico de Monterrey, Escuela de Ingeniería y Ciencias, Monterrey 64849, NL, Mexico; 2 Tecnologico de Monterrey, Centro de Biotecnología-FEMSA, Monterrey 64849, NL, Mexico; 3 Tecnologico de Monterrey, The Institute for Obesity Research, Monterrey 64849, NL, Mexico; 4 Laboratorio Nacional de Manufactura Aditiva y Digital MADiT, Apodaca 66629, NL, Mexico; 5 Centro de Investigación Numericalc, Monterrey 64000, NL, Mexico

**Keywords:** stereolithography, bioprinting, LCD, DLP, SLA, bioinks, UV, GelMA, low cost, cell culture, DIY

## Abstract

Light-based bioprinter manufacturing technology is still prohibitively expensive for organizations that rely on accessing three-dimensional biological constructs for research and tissue engineering endeavors. Currently, most of the bioprinting systems are based on commercial-grade-based systems or modified DIY (do it yourself) extrusion apparatuses. However, to date, few examples of the adoption of low-cost equipment have been found for light-based bioprinters. The requirement of large volumes of bioinks, their associated cost, and the lack of information regarding the parameter selection have undermined the adoption of this technology. This paper showcases the retrofitting and assessing of a low-cost Light-Based 3D printing system for tissue engineering. To evaluate the potential of a proposed design, a manufacturability test for different features, machine parameters, and Gelatin Methacryloyl (GelMA) concentrations for 7.5% and 10% was performed. Furthermore, a case study of a previously seeded hydrogel with C2C12 cells was successfully implemented as a proof of concept. On the manufacturability test, deviational errors were found between 0.7% to 13.3% for layer exposure times of 15 and 20 s. Live/Dead and Actin-Dapi fluorescence assays after 5 days of culture showed promising results in the cell viability, elongation, and alignment of 3D bioprinted structures. The retrofitting of low-cost equipment has the potential to enable researchers to create high-resolution structures and three-dimensional in vitro models.

## 1. Introduction

New methodologies for manufacturing biological tissues called 3D bioprinting are being employed in the field of tissue engineering to create sophisticated cellular structures for regenerative medicine, food printing, and in vitro models for research. Bioprinting uses microscale processes to assemble complex compound structures with bottom-up and top-down methods that can create complex structures by the controlled deposition of biomaterials in a layer-by-layer approach. There are several technologies reported in the literature whose taxonomy depends on the means to develop and manage the layers, including inkjet [1,2,3,4], extrusion [5,6,7], and Light-Based Bioprinting [8,9,10,11]. Each technique has distinct parameters and requirements, including resolution, printing speed, construct size, and printing temperature. The interaction of these parameters directly impacts the finesse available to reproduce a CAD sketch into truly three-dimensional constructs where cells can be placed and grow.

The tissues that make up the human body are composed of different kinds of cells. The artificial replication of these materials requires the balance of a complex trade-off between cost, setup accuracy, and processing parameters. Extrusion-Based Bioprinting (EBB) is the most common method due to the wide range of viscosities available to use as bioinks for manufacturing volumetric structures. The methodology is based on material deposition through mechanical or pneumatic-based nozzles. EBB has contributed to regenerative medicine projects, such as those concerning printed heart valves [12,13], skin tissue [14,15,16], cartilage [17,18], and blood vessels [7,19]. However, nozzles for 3D printing are associated with low printing resolution and the induction of damages by the tip. These processes discharge the material through a small output or nozzle (typically > 125 µm). Hence, when cells are integrated for culturing, shear stress produced during the deformation might impact their ability to grow and propagate [20]. Furthermore, reducing the nozzle size to achieve finer details reduces printability. There is an increasing number of commercial equipment designed for tissue engineering based on EBB, such as Cellink, Allevi, Envision Tec, and Organovo. This equipment features user-friendly interfaces and the ability for the samples to be sterilized, and the software includes customized processing parameters for bioinks. However, the ownership cost of these machines can be prohibitively high, and they are typically within a closed ecosystem of reagents and accessories.

Jet bioprinting (JB) includes a pioneering bioprinting approach that was created from the retrofitting of commercially available computer printers where the ink cartridge was replaced with biological material [21,22]. Drop-on-Demand (DoD) and Continuous inkjet printing (CIJ) are two subtypes of this type of technology. In the first one, a nozzle (typically with a diameter of 18 µm) ejects a droplet of material whenever the signal is given [23] to a thermal or piezoelectric printhead [24]. In the latter, the nozzle releases a constant flow of ink. Droplets are formed due to the Rayleigh–Plateau instability of the fluid [25]. The droplets are electrically charged and, with an electrical field, are sent to a specific location or target. Some of the applications for these technologies are creating bidimensional cell patterns to analyze the interactions between cells [26,27] and manufacturing three-dimensional biomimetic human skin tissue [14]. Nevertheless, since the manufacturing process is based on the passing of the bioink through a small output, shear stress may also cause a decrease in cell viability [28].

**Figure 1:**
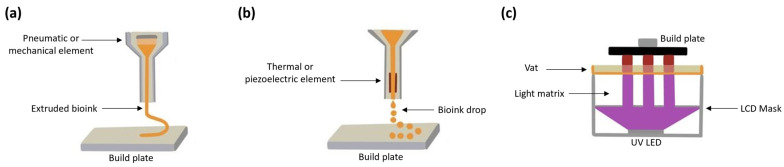
Bioprinting techniques: (**a**) Extrusion-Based Bioprinting, (**b**) Jet Bioprinting, and (**c**) Light-Based Bioprinting.

Light-Based Bioprinting (LBB) uses UV light to produce the cross linking of biomaterials and, at the same time, build a layer with a specific shape. This process has the advantage of having no mechanical stress since the bioink is contained in a reservoir with minimal interaction with other surfaces. The three previously described methods are presented in Figure 1.

**Figure 2:**
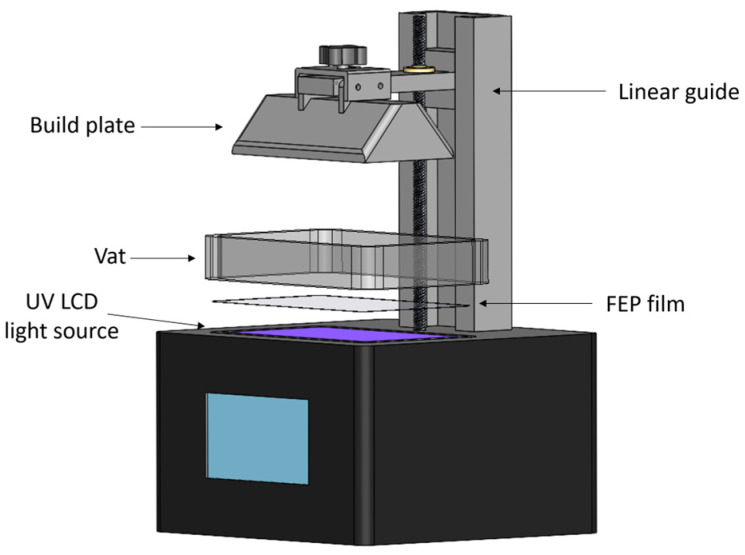
Main elements of an LCD 3D printer.

The light source, vat, and build plate are the three main parts of a light-based bioprinter (see Figure 2). The vat serves as a bioink reservoir tank. The build plate is a surface to which the planned construction is either directly attached or supported by a scaffold. Depending on the way the light interacts with a photocurable resin, different technologies are available: Stereolithography (SLA), Digital Light Projection (DLP), and Liquid Crystal Display (LCD). For LCD screen-based printers, the vat tank and the light source are often separated by an interface layer (Fluorinated Ethylene Propylene; FEP film in the Figure 2) that allows for an interaction with the light but inhibits polymerization on the bottom tank. Additionally, a pixel mask creates a two-dimensional figure to polymerize a layer with a specific pattern. In a printing process, the first polymerized layer must be firmly attached to the build plate to support the subsequent layers in a printed structure.

Commercially available bioprinters are expensive (between 16,000 and 25,000 USD) and have limited availability [29], and their prices can vary according to the functions, capabilities, and resolution. Consequently, many researchers are creating novel, affordable, and easy-to-use do it yourself (DIY) equipment. DIY bioprinters may incorporate 3D-printed parts, open-source firmware, and easy-to-acquire elements to build a complete and functional biomanufacturing system. These machines are focused on something other than implementing cutting-edge technology but can provide cheaper and more user-friendly procedures for research endeavors. The building cost of this equipment varies, but there is equipment that can be built with a low budget. The development of hardware and equipment as an enabler of the scientific community has been denominated as “open hardware” and has been a topic of interest for the community [30,31]. In Table 1, a list of some printers is provided for comparison.

Biomaterials are critical components for all types of bioprinting. To constitute the bulk of a tissue construct, several biomaterials can be used, such as Gelatin Methacryloyl (GelMA), Methacrylate Hyaluronic Acid (HAMA), and Polyethylene Glycol Diacrylate (PEGDA). In LBB, a photoinitiator must be used to promote photopolymerization (hardening of the bioink). The most common photoinitiators are Eosin Y, Triethanolamine (TEA), 1-Vinyl-2 Pyrrolidinone (NVP), or Lithium Phenyl -2, 4, 6-trimethylbenzoylphosphinate (LAP) [38]. The formulation of stable and high-resolution enabled photocurable biomaterials is a subject of interest for researchers. For example, combinations of different biomaterials and photoinitiators have been tested to generate specific-purpose bioinks [38]. One of the constraints of LBB bioinks is the scattering produced during cross linking in the presence of UV light. Photo inhibitors have been used as light filters for nonbiocompatible resins [39]. Unfortunately, typical photo inhibitors are usually toxic to cellular environments. Recent research on the employment of food dyes as photoinhibitors has reported an increase in the resolution of a process without compromising cellular viability [40]. Another limitation for researchers is that commercial photocurable biomaterials are expensive and the offered volumes are small (around 10 mL per sample). An additional challenge is the high volumes of bioink required to perform a single manufacturing process. Most commercially available light-based resin printers need high volumes of material to perform a 3D print (at least 300 mL of material). Considering that the bioinks are expensive and require effort and time to be synthesized, reducing the resin container to minimize the consumption of materials is necessary to develop more affordable systems.

There are many affordable 3D plastic resin printers that, with proper modifications, can be used as a biofabrication system. In a recent study, we demonstrated that modifying low-cost LBB additive manufacturing equipment to process biocompatible hydrogels is possible. In this work, we further optimize the lead times, precision, and repeatability of a microscale biomanufacturing process. To achieve these goals, we evaluated a (4K) LCD screen, redesigned critical system components (vat and build plate), and implemented bioink photoinhibition (color-controlled hydrogel). Furthermore, we implemented the insights to produce a previously cell-seeded hydrogel (C2C12) to create a 3D structure as a proof of concept to analyze the cell viability, elongation, and alignment that this biomanufacturing equipment could provide. The C2C12 cells are commonly used in the field of tissue engineering for in vitro metabolism tests and pharmaceutical development for current diseases [41].

## 2. Materials and Methods

### 2.1. GelMA Bioink Preparation

**Figure 3:**
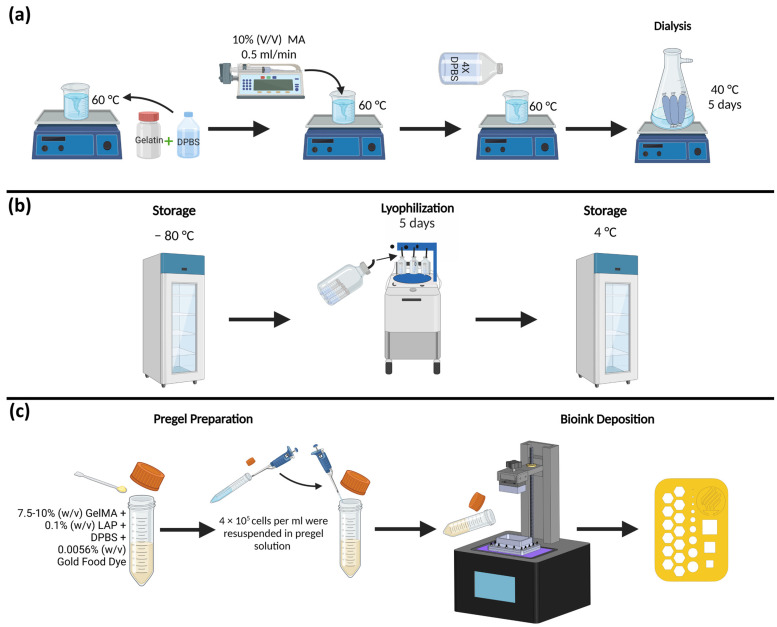
GelMA High synthesis deposition and testing steps: (**a**) compounds and processes used in GelMA synthesis; (**b**) lyophilization and storage phase; and (**c**) pregel preparation, cell seeding, bioink deposition, and example of the printed structure.

Type A porcine skin gelatin, methacrylic anhydride (MA), Dulbecco’s phosphate-buffered saline (DPBS), and Lithium Phenyl-2,4,6-trimethylbenzoylphosphinate (LAP) were purchased from Sigma Aldrich (MO, USA). The type A porcine skin gelatin was dissolved in the DPBS at 60 °C with a 10% (w/v) concentration. After homogenization, MA was added with a syringe pump at a flow rate of 0.5 mL/s at a concentration of 10% (w/v) to add the methacrylate substitution groups. To stop the reaction, the mixture was diluted with DPBS 4 times the total volume. A dialysis process of at least 5 days was performed at 40 °C using a dialysis tubing cellulose membrane with a pore size of 200 kDa. A lyophilization process was performed on the sample for 5 days and was stored at −80 °C until used. For the experimental study, batches of GelMA with concentrations of 7.5% and 10% were mixed with LAP (0.1% w/v) for photoinitiation. Since LAP is photosensitive, care was taken to handle the material in the dark. Gold Yellow food dye from ENCO was used in a concentration of 0.0056% (w/v) to prevent light scattering. Figure 3a–c describes the steps for the synthesis, deposition, and testing of GelMA.

### 2.2. 3D Printing Equipment

An Anycubic Photon Mono 4K (Anycubic, Shenzhen, China) 3D printer (3840 × 2400 pixels) was selected. The choice criteria among the alternatives were affordability, resolution, ease of use, and facility for changing components. The equipment cost was around 200 USD and is available worldwide. The nominal horizontal and vertical resolution were 35 µm and 10 µm, respectively. The rated output power density was 3.75 mW/cm^2^. This equipment also included a plastic resin tank with a capacity of 300 mL and an aluminum build plate to create structures with dimensions up to 165 × 132 × 80 mm (height × width × length).

A user interface with a 2.8-inch touchscreen was included to control the equipment, and a USB port was used to transfer the processed files in the slicer software Photon Workshop (V2.1.29). The printing parameters that can be changed for this 3D printer are Layer Height (L_H_), Normal Layer Exposure Time (L_T_), Bottom Layer Exposure Time (B_LT_), and Number of Bottom Layers (N_BL_).

### 2.3. Design and Manufacturing of Vat and Build Plate

A new tank for the biomaterial was designed using SolidWorks v2021 (Dassault Systémes SolidWorks, Velizy-Villacoublay, France). The reduced volume vat was composed of three elements: frame, FEP film, and enclosure. The frame was built in aluminum using a machining process. The enclosure was manufactured using benchtop SLA-LF 3D Form 3 (Formlabs, Somerville, MA, USA) additive manufacturing equipment, using a High Temp FLTHAM02 resin (Formlabs, Somerville, MA, USA). A 0.15 mm thick FEP film (ELEEGOO, Shenzhen, China) was cut with scissors and inserted between the frame and enclosure. A custom-made (low volume) plate was composed of a holder with the same SLA system as the enclosure, and the plate was built of aluminum.

### 2.4. Experimental Assessment for GelMA 3D Construct

**Figure 4:**
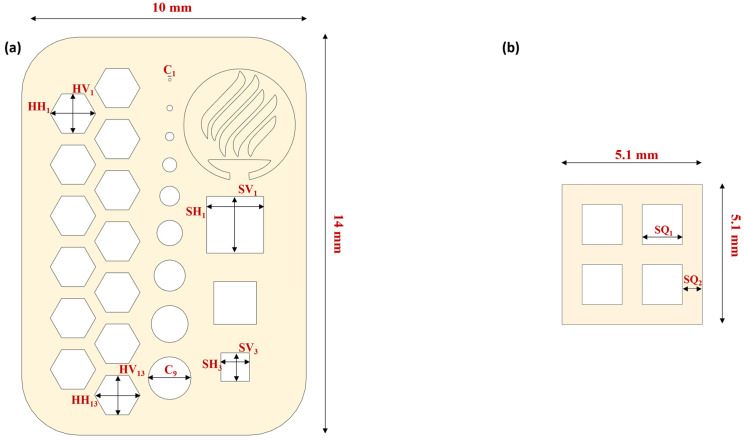
Resolution and cellular viability printing designs: (**a**) resolution and repeatability assessment design and (**b**) cellular viability assessment design.

An assessment of the printed structures was performed to measure the quality, reproducibility, and cell viability of the 3D-printed structures. A figure of 2 × 10 × 14 mm (height, width × length) with hexagonal, circular, and square micron-sized features and an 8 × 8 × 1 mm (width × length × height) (Figure 4) figure were designed using SolidWorks v2021 (Dassault Systémes SolidWorks, Velizy-Villacoublay, France). Measurements for the microfeatures of Figure 4a were made by using an AmScope stereoscopic microscope (United Scope L.L.C, Irvine, CA, USA) and a 12-megapixel AmScope camera connected to the microscope for the acquisition of images, and later the images were processed with AmScope software version 4.11.18573.

Table 2 summarizes the features evaluated during the assessment. The Bottom Layer Exposure Time (B_LT_), Number of Bottom Layers (N_BL_), and Layer Height (L_H_) were fixed for all the assessments to 45 s, 2 layers, and 50 µm, respectively. The printed structure had 40 layers in total. The structure was printed, varying the Normal Layer Exposure Time (L_T_) (10, 15, 20, 25, and 30 s). The experiment had three replicas (N = 3) and were evaluated for 7.5% and 10% GelMA concentrations. A total of 30 constructs were evaluated.

### 2.5. Cell Lines and Culture Medium

C2C12 myoblasts (CRL-1772) were acquired from the American Tissue Culture Collection (ATCC, Manassas, VA, USA). Briefly, early passage cells were cultured in a DMEM medium (Sigma-Aldrich, St. Louis, MO, USA) with a 10% fetal bovine serum (Gibco, ThermoFisher, Waltham, MA, USA) and 1% Anti-anti and Pen-Strep (ThermoFisher, Waltham, MA, USA) in a T75 cell culture flask (Corning Inc., Corning, NY, USA) under a humidified incubator (Sanyo, Osaka, JP) at 37 °C and 5% CO_2_. After the cells reached 75% confluence, subcultures were made using trypsin EDTA (Gibco, ThermoFisher, Waltham, MA, USA) 0.05% for 5 min. For the assessment, a 7 mL sample with 4 × 10^5^ cells/mL were prepared.

### 2.6. Actin-Dapi Staining

We washed the 3D-printed constructs three times with PBS 1X (ThermoFisher, Waltham, MA, USA). Then, the constructs were fixed with 4% paraformaldehyde (Sigma-Aldrich, St. Louis, MO, USA) for 1 h. Afterwards, we made two washes with PBS 1X and rinsed the constructs with DAPI 360 nm (Sigma Aldrich, St. Louis, MO, USA) and Phalloidin 647 nm (Abcam, Cambridge, UK) 3:1000 PBS 1X for one hour at 37 °C.

### 2.7. Cell Viability

Cell viability was evaluated using the Live/Dead kit (ThermoFisher, Waltham, MA, USA). To that aim, we washed the 3D-printed construct with a PBS 1X buffer (Thermo Fisher, Waltham, MA, USA). After 10 min, 50 µL of 2 µM calcein AM (final concentration) and 4 µM ethidium homodimer (EthD-1) were added to the constructs and were incubated at room temperature for 45 min. Constructs were visualized using a Zeiss Axio inverted microscope (Zeiss, Oberkochen, Germany Country) using GFP (green fluorescent protein) and RFP (red fluorescent protein) filters (Calcein AM 494/517 nm Ex/Em and ethidium homodimer-1 517–617 nm Ex/Em).

To quantify viability, we employed the open-source image processing software Fiji 2.9.0, which is a distribution of ImageJ with a bundle of scientific image analysis tools. To evaluate the number of live and dead cells, the images were segmented by color and size, and then the percentage of live cells was obtained [42].

## 3. Results and Discussion

The selected Light-Based 3D printing equipment had a light wavelength of 405 nm and a claimed power density of 3.75 mW/cm^2^. Both parameters were considered in the equipment selection to reduce cellular damage during the process. Furthermore, light power density is directly related to the lead times of the process. For the light wavelength, material photoinitiators worked in the range of 365 nm to 450 nm. UV radiation can affect the viability of cells; however, the rated power density of the retrofitted equipment was 3.5 mW/cm^2^, which is below other commercial equipment (i.e., Lumen X operates between 10 mW/cm^2^ and 30 mW/cm^2^). We selected LAP as our photoinitiator for this work, which has a peak light absorption at 405 nm. In this paper, we showcase the retrofitting of an Anycubic Photon Mono 4K to perform low-cost and high-resolution bioprinting. The following sections will present a detailed description of the hardware modifications to the additive manufacturing equipment and the evaluation of the machine’s capabilities.

### 3.1. 3D printing Equipment Retrofitting

The original resin container of the Anycubic 3D printer has a capacity of 300 mL of resin. In this work, we reduced the container volume to reduce the operation costs that filling the original tank would imply. The new container had a capacity of 30 mL, but could operate with 7.5 mL of photocurable bioink.

**Figure 5:**
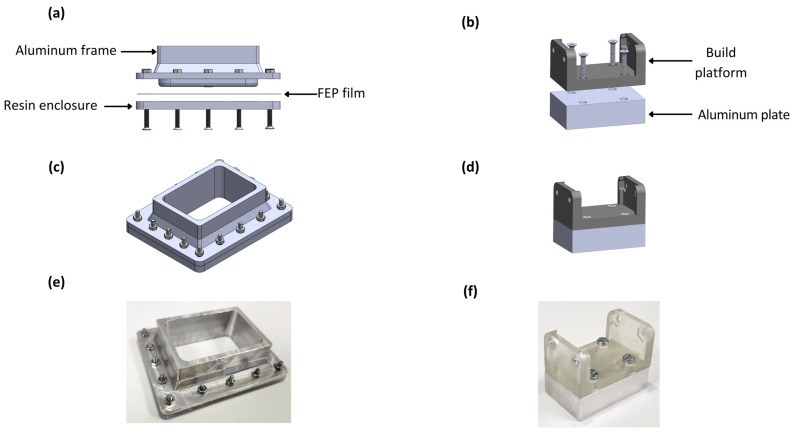
Elements of the bioprinting equipment retrofitting: (**a**) lateral exploded view of bioink container, (**b**) lateral exploded view of build plate, (**c**) isometric view of build plate, (**d**) isometric view of build plate, (**e**) manufactured vat, and (**f**) manufactured building plate.

As seen in Figure 5, the retrofitting consisted of two main elements: a modified vat and a build plate. In Figure 5a, the three main components of the vat are shown. The frame was manufactured in aluminum to facilitate cleaning the surface after each operation. The FEP film is a thin nonstick transparent surface that enables the passage of light and provides an impervious surface for the bioink. In Figure 5b, the retrofitted build plate is presented, consisting only of a resin build platform and an aluminum plate. The resin enclosure packages the elements with M3 stainless steel screws to seal the container, as seen in Figure 5c. The modified build plate had dimensions of 50 × 30 × 15 mm (length × width × height), and it was smaller than the vat superficial area to make the platform’s entrance possible.

All the modifications were performed to reduce the volume consumed in the process and make the bioprinter’s components reusable. As a result, the volume consumed in a single process was 90% lower than the volume that the usage of the original elements implied. The manufactured components are presented in Figure 5e,f.

### 3.2. Resolution and Repeatability Characterization

**Figure 6:**
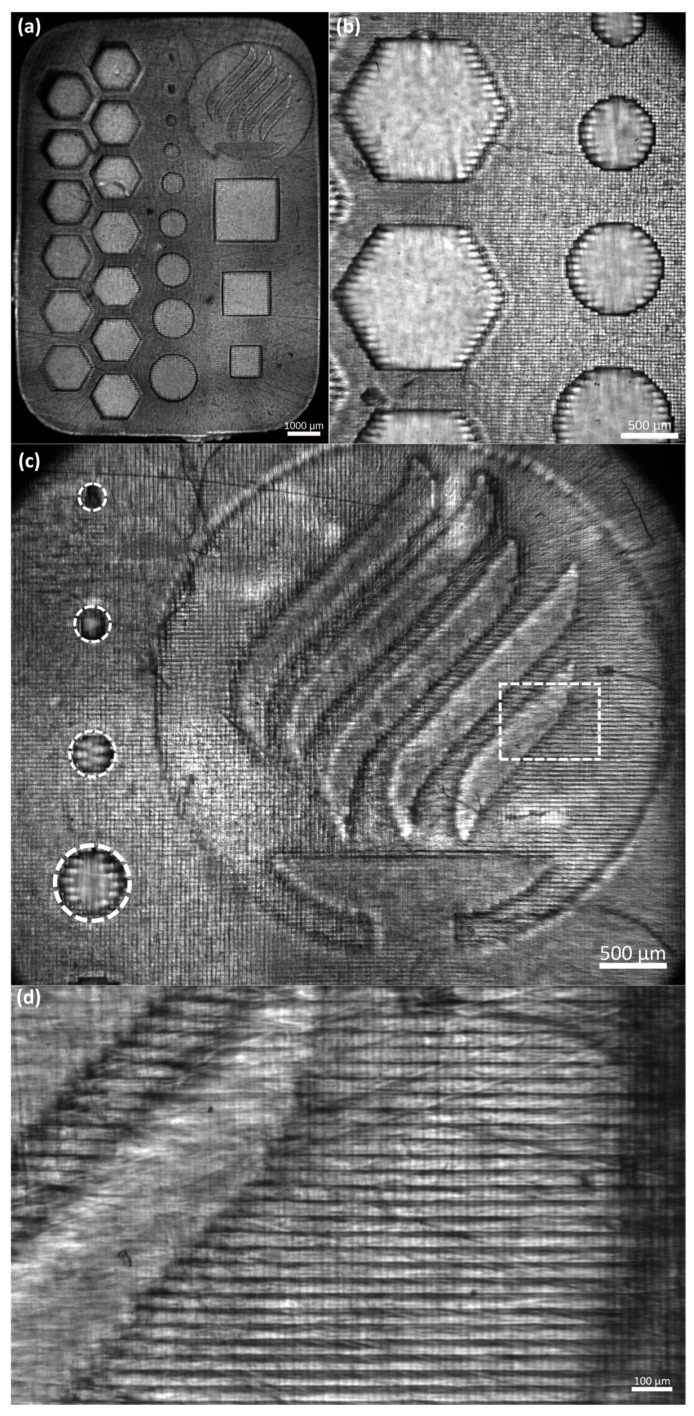
Microscope photo of manufacturing assessment for GelMA High 7.5%: (**a**) panoramic view; (**b**) hexagonal and circular features details; (**c**) Tecnologico de Monterrey with smallest circular features details (references in dotted white lines); and (**d**) horizontal lines details (referenced from dotted rectangle).

In this study, we used a single design to assess the resolution and repeatability of the equipment (see Figure 6a). The assessed structure was 2 × 10 × 14 mm (height × width × length) and contained features that could evaluate up to a 100-micron resolution. Table 2 presents the dimensions that were evaluated. Figure 6 shows the capacity of the printing equipment qualitatively. To assess the resolution capabilities of the printing equipment, we evaluated the deviations of the nominal values with the obtained dimensions of different concentrations and layer exposure times of the equipment, including the standard deviation. The mean absolute deviational error (MAE) for each feature was calculated following Equation (1):

(1) $$MAE\;feature=\frac{\left|Nominal-Experimental\right|}{Nominal}\times 100$$

The equation subtracts the obtained feature dimension length of the printed structure from the expected dimension of the uploaded sliced file. Then, the obtained value is normalized by dividing the subtraction with the nominal value and multiplying the result by 100 to obtain the percentage error.

Figure 6b presents details of the hexagonal and circular features. We can observe in more detail the repeatability, resolution, and capacity of creating patterned fibers between the hexagonal features. Another noticeable feature is that the printing presents horizontal lines that may affect the cell alignment during a bioprinted structure cell culture (see Figure 6d). Cell alignment is an essential parameter in the tissue engineering field, for example, in creating muscular cell patterns to mimic natural muscle fibers [43,44]. Creating these horizontal patterns where the cells can elongate to create fibers is one of the most remarkable features of this process. One possibility is that the LCD screen is the source of these patterns. As explained above, the LCD screen is a mask that enables the passage of light in a specific shape. The LCD screen is composed of thousands of pixels triggered to let the UV light pass in a designated pattern. Figure 6c presents the resolution of the equipment to print complex and miniaturized figures.

The Tecnologico de Monterrey logo is presented to show the capacity of the equipment to build detailed structures of different heights. The five curved lines form the flame, and the base of the cauldron has a height of 2 mm (40 layers). Meanwhile, the logo’s background (within the circle) has a height of 1 mm (20 layers). The smallest circular features go from 100 to 500 microns on the left side. In the case of the circular, hexagonal, and square features, we observed a membrane at the edges of the features. This phenomenon might be caused by the larger exposure time at the bottom layers. Further experimental research to characterize the features in a three-dimensional model is required.

**Figure 7:**
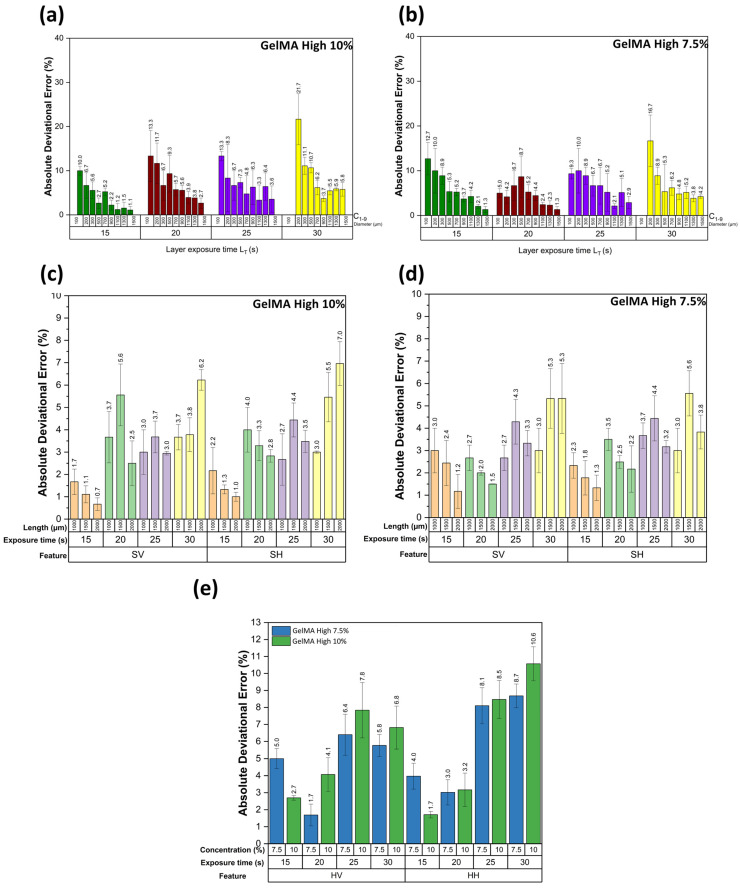
Absolute deviational error graphs of resolution and repeatability assessment figure: (**a**) circular features error at a concentration of 10% GelMA High, (**b**) circular features error at a concentration of 7.5% GelMA High, (**c**) square features error at a concentration of 10% GelMA High, (**d**) square features error at a concentration of 7.5% GelMA High, and (**e**) vertical and horizontal hexagonal features error.

In Figure 7, we present the results of the obtained deviational errors of each feature with its corresponding standard deviations. First, all the figures with a L_T_ of 10 s were not correctly printed. Therefore, this parameter was discarded. Figure 7a,b correspond to the circular features with a GelMA High concentration of 10% and 7.5%, respectively; we can observe that the L_T_ of 15 and 20 s presented a lower error than the other printing times. An increase in the error in the smallest features was expected, the characteristics of 100 microns were inconsistent, and the shape was not a perfect circle, as seen in Figure 6c. In the case of the features of 200 microns, we can observe that the error was decreased. Another important observation is that the standard deviation of the results was increased when the L_T_ was also increased. The features of 100 microns were not printed for an L_T_ of 30 s due to overexposure to light.

Figure 7c,d correspond to the square features with a GelMA High concentration of 10% and 7.5%, respectively; in both cases, the L_T_ of 15 s presented the lowest error ratio compared to the other exposure times. Vertical and horizontal errors are similar at the exact exposure times in both concentrations. It was observed that there were significant differences in the error presented in the L_T_ of 15 s in comparison to the other exposure times. In the L_T_ 15 and 20 s, the error was reduced when the feature size was increased, which did not seem to happen in the other exposure times. For the hexagonal features, we obtained the average error of the 13 hexagonal features to characterize the repeatability of the printing; Figure 7c shows that the L_T_ of 15 and 20 s presented the lower errors as shown in the circular and square features. In the vertical and horizontal hexagonal features with the L_T_ of 15 s with a concentration of 10% GelMA High, we observed that the values were significantly different from the other exposure times, as seen in Figure 7e.

### 3.3. Biological Characterization

**Figure 8:**
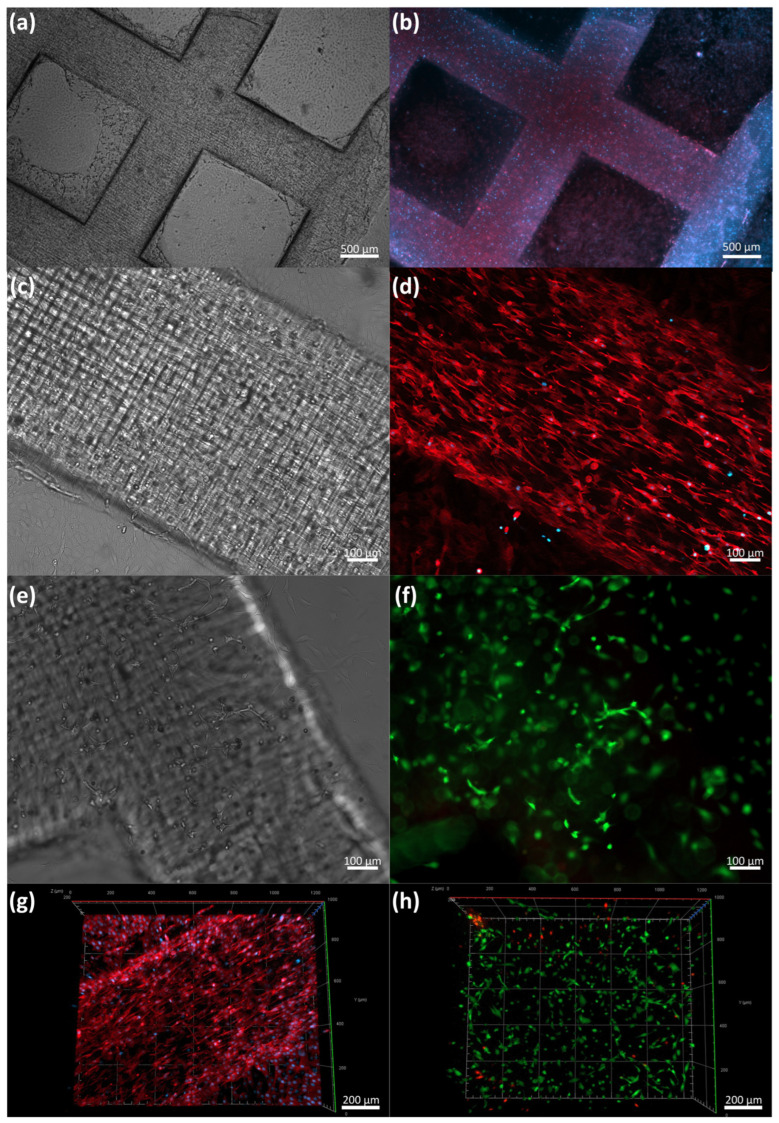
Proof-of-concept cell seeding: (**a**) bright field image of 3D-printed GelMA (10%) construct laden with C2C12 after 5 days of culture, (**b**) Actin-Dapi staining of whole construct, (**c**) detail of bright field image of 3D-printed GelMA (10%) construct laden with C2C12 after 5 days of culture, (**d**) detail of of Actin-Dapi staining (**e**,**f**) bright field and Live/Dead fluorescence assay after 5 days of culture, and (**g**,**h**) orthogonal reconstruction of Z-stack images of Actin-Dapi staining and Live/Dead fluorescence assay.

As proof of concept of the low-cost LBB for cell culture, we manufactured the construct described in Figure 4b (dimensions are summarized in Table 2). Figure 8 shows fluorescence and bright field micrographs at different culture times.

For this set of experiments, we used GelMA 10% w/v as a printing hydrogel. GelMA is a synthetic polymer produced from the hydrolysis and denaturation of collagen. These samples had an arginine-glycine-aspartic-acid sequence (RGD), enhancing cell adhesion, proliferation, and differentiation. The L_T_ used in this process was 15 s and the total printing time was 10 min. We observed that the C2C12 cells started to spread and elongate during the first 24 h after printing.

The cells began to align on day 3 in the construct as the culture times increased. We performed a Live/Dead assay to estimate the cell viability; as expected, most cells were alive after the first five days of printing, with 93.01% of the cells alive, suggesting that the process was gentle and benign for cells. Importantly, we observed spread and alignment in the whole construct after the first three days. Actin-DAPI Z stack micrographs of the construct on day 5 demonstrated the alignment and growth of C2C12 in the printed construct. The spontaneous cell alignment in our constructs was probably due to the printing and characteristics of the UV light LCD mask emission system. We anticipated that the process would exhibit align patterns in the internal surfaces, which boosted cell polarization and alignment.

## 4. Conclusions

We successfully employed a low-cost 3D additive manufacturing system retrofitting strategy for bioprinting. The design of a new vat tank reduced 90% of the required volume of bioink, providing an alternative to producing biological structures with smaller batches.

Considering that this is very affordable equipment, the redesigned 3D printer was able to achieve high-quality hydrogel-based constructs. On the manufacturability test, deviational errors were between 0.7% to 21.7%. While an exposure time of 10 s could not be applied for a successful bioprinting process, 15 and 20 s presented the smallest deviational error (0.7% to 13.3%). The total printing time for the constructs was approximately 10 min. Considering the characteristics of the LCD screen, up to four designs could be printed simultaneously without additional hardware or software modifications. However, upscalability should be addressed in future work.

We successfully bioprinted muscular C2C12 cells using GelMA as the bioink. The proof of concept exhibits that this retrofitted system can be used as a low-cost bioprinting platform. The findings presented here provide a starting point for further examinations of different types of cells and photoinhibitors.

## Figures and Tables

**Table 1:** Bioprinting equipment. Examples of 3D bioprinting equipment, technique, resolution, and costs. Adapted from [32].

Printer Name	Manufacturing Technique	Type *	Resolution	Cost (USD)
Multimaterial 3D bioprinter [33]	Jet based	E	Not available	650
DIY Bioprinter [34]	Extrusion based	E	~100 µm	300
High-Resolution SLA bioprinting system [8]	Light based	E	50 µm	1500
Smartphone-enabled DLP printer [31]	Light based	E	50 µm	1000
Computed Axial Lithography (CAL) volumetric [35]	Light based	E	200 µm	4000
Tissuelabs TissueRay [36]	Light based	C	150 µm	16,000
Cellink Lumen X [37]	Light based	C	35 µm	25,000

* E = experimental, C = commercial.

**Table 2:** Features for bioprinting resolution assessment.

Type of Feature	Symbol	Dimensions (µm)
Hexagonal Vertical Length (HV)	HV_1-13_	1600
Hexagonal Horizontal Length (HH)	HH_1-13_	1390
Circular Diameter (C)	C_1-9_	100, 200, 300, 500, 700, 900, 1100, 1300, and 1500
Square Vertical Length (SV) and Square Horizontal Length (SH)	SV_1-3_ and SH_1-3_	1000, 1500, and 2000
Square Dimensions	SQ_1-2_	1500 and 700

## Data Availability

The data presented in this study are openly available in FigShare at 10.6084/m9.figshare.21761075.
